# Cobalt Resistance via Detoxification and Mineralization in the Iron-Reducing Bacterium *Geobacter sulfurreducens*

**DOI:** 10.3389/fmicb.2020.600463

**Published:** 2020-11-26

**Authors:** Hunter Dulay, Marcela Tabares, Kazem Kashefi, Gemma Reguera

**Affiliations:** Department of Microbiology and Molecular Genetics, Michigan State University, East Lansing, MI, United States

**Keywords:** cobalt detoxification, metal homeostasis, stress response, extracellular electron transfer, cytochromes, biomineralization

## Abstract

Bacteria in the genus *Geobacter* thrive in iron- and manganese-rich environments where the divalent cobalt cation (Co^II^) accumulates to potentially toxic concentrations. Consistent with selective pressure from environmental exposure, the model laboratory representative *Geobacter sulfurreducens* grew with CoCl_2_ concentrations (1 mM) typically used to enrich for metal-resistant bacteria from contaminated sites. We reconstructed from genomic data canonical pathways for Co^II^ import and assimilation into cofactors (cobamides) that support the growth of numerous syntrophic partners. We also identified several metal efflux pumps, including one that was specifically upregulated by Co^II^. Cells acclimated to metal stress by downregulating non-essential proteins with metals and thiol groups that Co^II^ preferentially targets. They also activated sensory and regulatory proteins involved in detoxification as well as pathways for protein and DNA repair. In addition, *G. sulfurreducens* upregulated respiratory chains that could have contributed to the reductive mineralization of the metal on the cell surface. Transcriptomic evidence also revealed pathways for cell envelope modification that increased metal resistance and promoted cell-cell aggregation and biofilm formation in stationary phase. These complex adaptive responses confer on *Geobacter* a competitive advantage for growth in metal-rich environments that are essential to the sustainability of cobamide-dependent microbiomes and the sequestration of the metal in hitherto unknown biomineralization reactions.

## Introduction

Metal micronutrients such as nickel (Ni^II^), cobalt (Co^II^), manganese (Mn^II^), and iron (Fe^II^) are essential for life yet toxic above relatively low concentrations (Buccella et al., 2019). Not surprisingly, microorganisms have evolved numerous adaptive responses to import the essential metals from the environment while preventing their excessive intracellular accumulation and intoxication (Chandrangsu et al., 2017). Metal homeostasis is primarily achieved by the antagonistic activities of metal importers and exporters (Chandrangsu et al., 2017). Cells often use high affinity transporters to import the metals with specificity and rely on specialized proteins and chaperones to integrate them into pathways dedicated to the synthesis of metalloproteins and enzyme cofactors (Buccella et al., 2019). Collectively, biometals contribute to the synthesis of up to one third of the cell’s proteome and to metabolic functions essential to the growth and survival of the cell (Buccella et al., 2019). Each of these metals must be available in just the right intracellular concentration (i.e., the cellular metal quota) to prevent intoxication (Outten and O’Halloran, 2001). Thus, dedicated metalloregulatory systems monitor the intracellular metal levels and modulate the expression of transporters and other proteins essential for metal homeostasis (Chandrangsu et al., 2017). Metal exporters provide the primary mechanism to eliminate excess metal (Chandrangsu et al., 2017) but the cellular response to metal intoxication is often more extensive, as cells have to cope with the direct and indirect impacts of the reactive metals on proteins and DNA. For example, Co^II^ can bind and inactivate numerous proteins non-specifically, displace other metals (particularly, Fe^II^) from prosthetic groups and metal-binding sites, and generate free radicals (Valko et al., 2005). Its high affinity for thiol groups disrupts disulfide bonds in proteins, reduces the free thiol pool and can interfere with sulfur assimilation (Barras and Fontecave, 2011). Hence, Co^II^ intoxication causes generalized damage in the cells, requiring extensive reprogramming to cope with multiple stressors.

The essentiality yet toxicity of metal micronutrients such as Co^II^ exerts selective pressure on microorganisms to tune their metabolism to the fluctuating availability of the metal species from geochemical sources. Yet, many aspects of the biological cycling of metal micronutrients remain relatively obscure. This is particularly true for Co^II^, a metal micronutrient that some microorganisms assimilate to produce enzyme cofactors (cobamides) in the cobalamin (vitamin B_12_) family involved in metabolic reactions essential to all living cells (Shelton et al., 2019). Genes encoding cobamide-dependent enzymes are widespread in prokaryotes but only a fraction of surveyed genomes have complete pathways for *de novo* cobamide synthesis (Zhang et al., 2009; Shelton et al., 2019). As a result, most microorganisms need to salvage cobamides from the environment, a nutritional dependency that drives syntrophic interactions with cobamide producers (Seth and Taga, 2014). Cobamide-dependent microbiomes depend on the ability of cobamide producers to import and assimilate the soluble Co^II^ cation. The divalent species, however, readily oxidizes to Co^III^ on the surface of Mn^IV^ oxide particles (Crowther et al., 1983; Kay et al., 2001). Co^II^ mobility in soil and sediment systems is also limited by the tendency of the metal to coprecipitate with Fe^III^ and Mn^IV^ oxide minerals (Krupka and Serne, 2002). Additionally, Fe^III^ and Mn^IV^ oxides sorb large amounts of the metal cation, sequestering it in solid phases that reduce its bioavailability (Backes et al., 1995).

**FIGURE 1:**
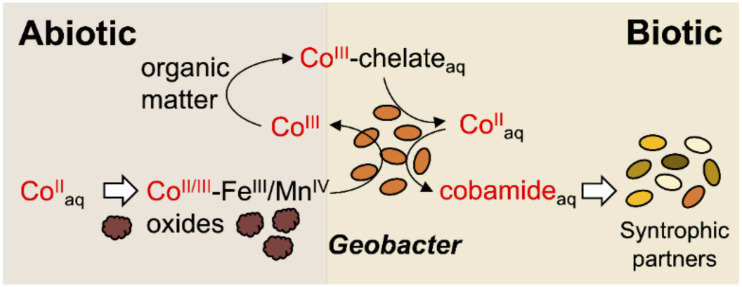
Known contribution of *Geobacter* species to the cycling of cobalt (Co). *Geobacter* bacteria reduce chelated and mineral forms of Co^III^ to Co^II^, whose bioavailability is limited by the tendency of the Co species to adsorb and/or co-precipitate with Fe^III^ and Mn^IV^ oxides. The reduction of Fe^III^ and Mn^IV^ oxides by *Geobacter* bacteria solubilizes Co^II^ for the synthesis of cobamides that support the growth of syntrophic partners.

The absorption and co-precipitation of most of the available Co^II^ into Fe^III^ and Mn^IV^ minerals gives Fe^III^ and Mn^IV^-reducing bacteria, such as those in the genus *Geobacter*, a competitive advantage for growth in cobamide-dependent microbiomes (Figure 1). These bacteria gain energy for growth from the reductive dissolution of the metal oxides, which are reactions that solubilize Fe^II^ and Mn^II^ and remobilize Co^II^ and Co^III^ (Reguera and Kashefi, 2019). *Geobacter* species are also important drivers of organic matter degradation, a process that generates organic chelators with affinity for Co^III^. This keeps the trivalent species in solution and available for use as an electron acceptor (Reguera and Kashefi, 2019). The dissimilatory reduction of chelated forms of Co^III^ by *Geobacter* reduces Co^III^ to Co^II^ (Reguera and Kashefi, 2019). The low reduction potential of the Co^II^ species (−0.28 V versus standard hydrogen electrode, SHE) and its toxicity to bacteria at relatively low concentrations have been assumed to prevent its biological reduction to Co^0^ (Hau et al., 2008; Cosert and Reguera, 2019). Despite its toxicity, *Geobacter* species, including the model laboratory strain *Geobacter sulfurreducens*, assimilate Co^II^ to synthesize cobamides, which they secrete to sustain several syntrophic partners (Yan et al., 2012) (Figure 1). These syntrophic interactions are favored in local epigenetic zones enriched in Fe^III^ and Mn^IV^ oxides, where Co^II^ preferentially accumulates (Burkhardt et al., 2009). This raises yet unexplored questions about the cellular tolerance of *Geobacter* species for Co^II^ and the mechanisms that allow these microorganisms to survive and even thrive in Co^II^-rich environments.

We gained insights into the environmental controls of *Geobacter* activities in cobamide-driven microbiomes by investigating the adaptive responses of *G. sulfurreducens* to growth and reproduction in the presence of Co^II^. Consistent with environmental exposure, we demonstrate high Co^II^ resistance in this laboratory strain and describe pathways for protein and DNA repair, cell envelope modifications, and biofilm formation that allow the cells to effectively cope with Co^II^ stress. Importantly, we show that metal acclimation activates respiratory chains that could participate in the reductive precipitation of the metal on the cell’s surface to alleviate toxicity. These adaptive responses allow *Geobacter* species to grow in Co^II^-rich environments, sustaining the productivity of the native microbiomes and contributing to hitherto unknown reactions of the Co cycle.

## Results

### Genomic Determinants of Co^II^ Homeostasis in *G. sulfurreducens*

**FIGURE 2:**
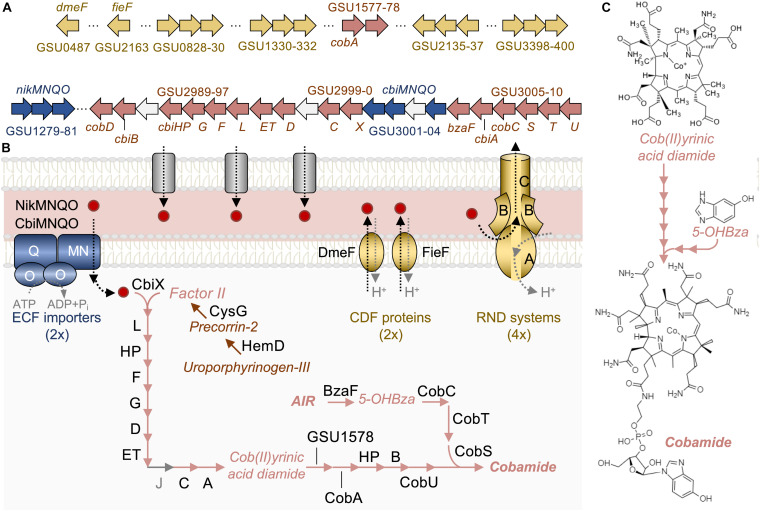
Genomic reconstruction of potential pathways for Co^II^ transport and assimilation in *G. sulfurreducens*. The genes **(A)** and model **(B)** show transmembrane ECF importers (in blue) as well as two CDF proteins for transmembrane export and four RND systems for exporting periplasmic metal across the outer membrane (gold color). Panel **(B)** also shows generic outer membrane porins (in gray) for the simple diffusion of metal cations. The genome of *G. sulfurreducens* also encodes for Cbi (letter designation), Cob (full name) and other enzymes needed for the anaerobic synthesis of cobamide (pink arrows). The pathway starts with the incorporation by the cobaltochelatase CbiX of the metal into Factor II, which is synthesized from heme intermediates such as uroporphyrinogen-III. The genome encodes Cbi proteins that convert the Co^II^-Factor II into cob(II)yrinic acid diamide, except for CbiJ (in gray) whose role in the pathway is yet to be experimentally validated. Cob (full name) and Cbi (only letters) proteins and other enzymes complete the synthesis of a cobamide with a 5-OHBza lower ligand (formula in **C**).

Metal ions bridge the outer membrane of Gram-negative bacteria by simple diffusion through nonselective pores (Nikaido, 2003) but require specific transporters to traverse the inner membrane (Figure 2). We identified in *G. sulfurreducens* complete NikMNQO and CbiMNQO importers, the most widespread prokaryotic systems for Ni^II^ and Co^II^ uptake (Rodionov et al., 2006). Although both systems can import Ni^II^ and Co^II^, specific amino acid signatures in the M subunits make CbiMNQO the high affinity importer of Co^II^ (Rodionov et al., 2006). At high enough concentrations, however, Co^II^ could selectively outcompete Ni^II^ and enter the cytoplasm via the NikMNQO system. These importers are annotated as ATP-binding Cassette (ABC) transporters, but they are part of the prokaryotic family of energy-coupling factor (ECF) systems that also transport water-soluble vitamins and cofactors (Cracan and Banerjee, 2013). The metal ECF subclass has a distinct modular architecture (A, T, and S components) to bind the substrate (S component) without assistance from extracytoplasmic solute-binding proteins (Cracan and Banerjee, 2013). In most Nik/CbiMNQO systems, the S component is a heterodimer of M and N subunits (Rodionov et al., 2006) but these subunits are fused in a single gene in *G. sulfurreducens* (*nikMN*, GSU1279; *cbiMN*, GSU3004). The end result is the same: the assembly of a MN subcomplex (S component) that binds the metal and transports it across the membrane in a reaction energized by the O subunit dimer (A, or ATPase, component) and rate-modulated via interactions with the transmembrane Q subunit (T component) (Cracan and Banerjee, 2013).

As shown in Figure 2, the *cbiMNQO* genes are part of a large cluster (GSU2989–3010) encoding most of the enzymes needed for the anaerobic synthesis of a cobinamide intermediate (Cbi proteins) and its conversion into cobamide (Cob and Cbi proteins) (Moore and Warren, 2012). We identified in a separate genomic location two additional cobamide biosynthetic enzymes, GSU1578 and CobA (GSU1577). Also unlinked were two genes encoding enzymes for the methylation (HemD, GSU3286) and oxidation (CysG, GSU3282) of uroporphyrinogen III, the common precursor of cobamide and heme biosynthesis (Moore and Warren, 2012). These two enzymes convert uroporphyrinogen III into Factor II, the preferred substrate for the anaerobic synthesis of cobamide (Moore and Warren, 2012). The anaerobic cobaltochelatase CbiX (GSU3000) incorporates the metal into Factor II, while several Cbi proteins methylate, contract, amidate, and decarboxylate the molecule to generate a cobyrinic acid diamide intermediate (Figure 2C). All of the proteins needed for these reactions were annotated or had a clear homolog in the genome of *G. sulfurreducens*, except for the precorrin-6X reductase CbiJ (highlighted in gray in Figure 2B). This enzyme is often assigned to this reaction based on its homology with the aerobic enzyme CobK, but its biological role has never been confirmed (Moore and Warren, 2012). The cob(II)yrinic acid diamide intermediate is then converted into adenosyl cobinamide in sequential reactions initiated by an adenosylcobamide-binding subunit of an (R)-methylmalonyl-CoA mutase (GSU1578). The step catalyzed by CobU (GSU3010) generates an adenosine-GDP-cobinamide substrate for attachment of the cobamide lower ligand. *G. sulfurreducens* produces a cobamide with a 5-hydroxybenzimidazole (5-OHBza) lower ligand (Figure 2C) that is synthesized from 5-amino-imidazole ribonucleotide (AIR) by BzaF (GSU3005) (Hazra et al., 2015). The *bzaF* gene, which is unique to the *Geobacteraceae* and other members of the order *Desulfurococcales*, is a functional homolog of the *bzaA and bzaB* genes that catalyze the synthesis of 5-OHBza in other bacteria (Hazra et al., 2015). The attachment of the lower ligand to adenosine-GDP-cobinamide completes the synthesis of the cobamide (Figure 2C).

At high enough concentrations, Co^II^ can also enter the inner membrane non-specifically via magnesium (Mg^II^) uptake systems (Nies, 1999). To compensate for the uncontrolled influx of the metal, cells express metal exporters (Chandrangsu et al., 2017). We identified in the genome two genes (GSU0487 and GSU2613) encoding the cation diffusion facilitator (CDF) proteins, DmeF and FieF (Figure 2). These proton-driven antiporters export a broad range of divalent cations (Co^II^, Zn^II^, Fe^II^, Cd^II^, and Ni^II^) across the inner membrane (Kolaj-Robin et al., 2015). However, the preferred substrate for DmeF is Co^II^ (Munkelt et al., 2004) while FieF specializes in the export of excess Fe^II^ (Grass et al., 2005). The intracellular accumulation of Co^II^ can disrupt the homeostatic balance with Fe^II^ and allow FieF to move more Co^II^ than Fe^II^ across the inner membrane. The genome also contains four tripartite metal efflux systems of the Resistance-Nodulation-Division (RND) superfamily (Munkelt et al., 2004) (Figure 2). RND efflux pumps use the proton gradient to energize the export of cytoplasmic or periplasmic substrates (Kim et al., 2011). Some of these exporters function as multidrug efflux pumps (Nikaido and Takatsuka, 2009) while others specialize in proton-dependent transport of divalent metal cations (Bagai et al., 2008; Pak et al., 2013). As in other Gram-negative bacteria (Nikaido and Takatsuka, 2009), the *Geobacter* metal RND systems contain a transmembrane pump, a periplasmic adaptor protein and an outer membrane porin. This trimeric configuration facilitates the export of periplasmic metals using the electrochemical gradient (Nikaido and Takatsuka, 2009). Collectively, inner and outer metal exporters ensure that Co^II^ does not accumulate to toxic levels inside the cytoplasm (Chandrangsu et al., 2017).

### High Co^II^ Tolerance in *G. sulfurreducens* Suggests Significant Environmental Exposure to the Metal

**FIGURE 3:**
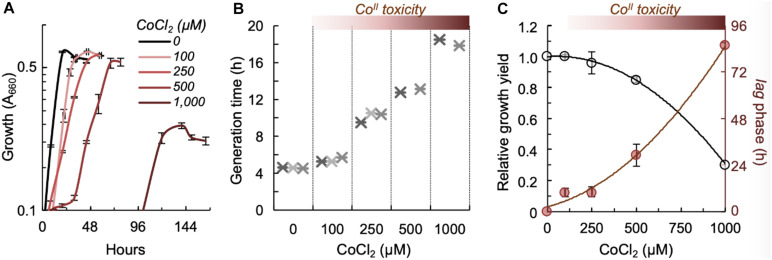
Effect of Co^II^ on *G. sulfurreducens* growth. **(A)** Cell growth (absorbance at 660 nm) in acetate-fumarate cultures with or without CoCl_2_ supplementation (up to 1,000 μM). Data points for 0–250 μM CoCl_2_ treatments show average and standard deviation of triplicate cultures. Treatments with 500 and 1,000 μM CoCl_2_ show average and standard error of the only two replicates that resumed growth within a week. **(B,C)** Effect of Co^II^ toxicity on growth efficiency. Panel **(B)** shows generation (doubling) times for each of the replicates in the untreated (0 μM) and treated (100–1,000 μM) cultures shown in **(A)**. Panel **(C)** shows the effect of the CoCl_2_ treatment in reducing the culture’s growth yields (OD_660_ in early stationary phase relative to the untreated 0 μM cultures) or in extending the *lag* phase (time before entry in exponential phase). The trendlines in **(C)** are the polynomial fits for the average data points of relative growth yields (*R*^2^ = 0.999) and *lag* phases (*R*^2^ = 0.994) from the cultures shown in **(A)**.

As metal resistance evolves under selective pressure, we determined the growth efficiency of *G. sulfurreducens* in the presence of Co^II^ (Figure 3). For these experiments, we inoculated cells at low densities (OD_660_, ∼0.03) in a medium optimized for growth of *G. sulfurreducens* (DB medium) (Speers and Reguera, 2012) with acetate and fumarate (DBAF medium) and supplemented with various concentrations of CoCl_2_. We measured growth with up to 1 mM CoCl_2_ (Figure 3A), a concentration commonly used to enrich for metal-resistant bacteria from soils and industrial wastes contaminated with heavy metals (Schmidt and Schlegel, 1989). Generation times increased (Figure 3B) and planktonic biomass yields (maximum OD_6__6__0_ during entry in stationary phase) decreased (Figure 3C) in a dose-dependent manner, as cells coped with higher levels of metal toxicity. For example, *G. sulfurreducens* cells doubled every 4.58 (± 0.05) hours in the untreated cultures, which we estimated to have approximately 27 μM of Co^II^ using an assay we developed for the colorimetric detection of the metal in the culture medium. Supplementation with an additional 100 or 250 μM CoCl_2_ increased generation times to 5.41 (± 0.25) and 10.13 (± 0.54) hours, respectively (Figure 3B). Generation times increased even more at higher CoCl_2_ concentrations (500 and 1,000 μM) and, on average, one out of three replicate cultures did not resume growth after a week (Figure 3B). Furthermore, cultures that resumed growth did so after extended phases of acclimation (long *lag* phases before entering exponential phase) and reached lowest biomass yields (Figure 3C).

### Transcriptomic Analysis Reveals Multiple Mechanisms for Co^II^ Detoxification

**FIGURE 4:**
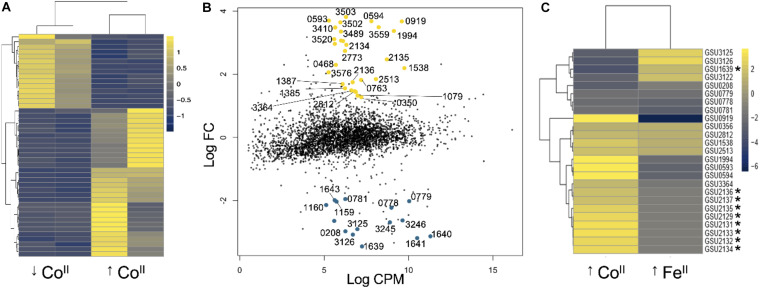
Transcriptional response of *G. sulfurreducens* to Co^II^. **(A)** Heatmap of the transcriptional response in two replicates for the untreated (↓Co^II^) and treated (↑Co^II^) cultures. The datasets and statistical analyses of the expression data are provided in Supplementary Data 1. **(B)** Dispersion plot of transcript abundance (log fold change [*log*FC] versus log counts per million [*log*CPM]) identifying the significantly upregulated (yellow) and downregulated (blue) genes. **(C)** Heatmap of genes differentially expressed with excess Co^II^ or Fe^II^. The latter was calculated as the inverse ratio of the log_2_ fold transcriptional changes reported for *G. sulfurreducens* cultures growing with sufficient versus excess Fe^II^ (Embree et al., 2014). The asterisk shows genes under Fur control (Embree et al., 2014). The calculations and data comparisons are provided in Supplementary Data 2.

We gained insights into the mechanisms that allow *G. sulfurreducens* to cope with Co^II^ stress by comparing the transcript abundance of mid-log phase cells grown with or without 250 μM CoCl_2_ supplementation (Figure 4). Co^II^ stress led to the differential expression of 47 genes. Of them, 32 were upregulated (Table 1) and 15 were downregulated (Table 2). This is approximately 0.9% (upregulated) and 0.4% (downregulated) of the genes annotated in the genome of *G. sulfurreducens*. Most of the upregulated genes encoded proteins with predicted roles in metal detoxification such as efflux pumps, protein and DNA repair enzymes, cell envelope modification pathways, and transcriptional regulation (Table 1). We also identified among the upregulated genes pathways for extracellular electron transfer that could provide a mechanism for energy transduction and Co^II^ mineralization on the cell surface. By contrast, most of the downregulated genes coded for non-essential proteins with metal-binding domains or amino acids that Co^II^ is known to bind strongly (Table 2). Thus, their downregulation reduces the burden of Co^II^ retention in the cell’s proteome.

**TABLE 1:** Upregulated genes in cobalt transcriptome.

Function (no. of genes)	Locus	Gene	Gene product	log(2) FC	Metal-binding motif (Pfam)	Subcellular localization
Transmembrane transport (3)	GSU2135	*czcA*	CusA/CzcA heavy Metal efflux RND transporter	2.47		Inner membrane
	GSU2136	*czcB*	Efflux RND transporter, periplasmic adaptor subunit	1.75		Periplasm
	GSU2137	*czcC*	Outer membrane pore/TolC family protein	1.69		Outer membrane
Electron transfer (4)	GSU0593	*cbcB*	Cytochrome *b*, putative	3.70	Prokaryotic cytochrome *b561* (PF01292)	Inner membrane
	GSU0594	*cbcA*	Cytochrome *c*, heptaheme	3.68	Doubled CXXCH motif (PF09699)	Periplasm (membrane-bound)
	GSU1538		Cytochrome *c* peroxidase	2.19	Di-haem cytochrome *c* peroxidase (PF03150)	Periplasm
	GSU2513		Cytochrome *c*, monoheme	1.85	Cytochrome *c* oxidase, *cbb3*-type (PF13442)	Periplasm
Type-I CRISPR-Cas system (2)	GSU1385	*cse1*	CRISPR processing complex protein CasA	1.56	CRISPR_Cse1 (PF09481)	Cytoplasm
	GSU1387	*cse4*	CRISPR processing complex protein CasC	1.69		Cytoplasm
Cell redox homeostasis (1)	GSU2812		Glutaredoxin family protein	1.44		Periplasm
Cell envelope (4)	GSU1079		PEP motif-containing protein, putative exosortase substrate	1.32		Extracellular
	GSU1994		PEP motif-containing protein, putative exosortase substrate	3.37		Extracellular
	GSU2133		Lipoprotein	3.07		Non-cytoplasmic
	GSU3576		Lipoprotein, putative	2.06		Outer membrane
Signal transduction (3)	GSU0356		Sensor histidine kinase, heme-binding	1.27	Heme-binding (PF11845)	Inner membrane
	GSU2134		P-II family nitrogen regulator	2.93		Cytoplasm
	GSU3364	*hgtR*	Hydrogen-dependent growth transcriptional repressor	1.49		Cytoplasm
DNA repair (1)	GSU0763		Helicase, putative	1.82		Cytoplasm
Transposon functions (1)	GSU2772		Transposase of ISGsu3, IS5 family	1.46		
Unknown function (13)	GSU0468		Hypothetical protein	2.30		Inner membrane
	GSU0919		Hypothetical protein	3.67		Unknown
	GSU0959		Hypothetical protein	1.61		Cytoplasm
	GSU2129		Hypothetical protein	3.49		Non-cytoplasmic
	GSU2131		Hypothetical protein	3.05		Non-cytoplasmic
	GSU2132		Hypothetical protein	2.99		Unknown
	GSU2773		Hypothetical protein/ATP-dependent Clp protease proteolytic subunit	2.74		Unknown
	GSU3410		Hypothetical protein	3.48		Inner membrane
	GSU3489		Hypothetical protein	3.35		Inner membrane
	GSU3502		Hypothetical protein	3.64		Inner membrane
	GSU3503		Hypothetical protein	3.81		Cytoplasm
	GSU3520		Hypothetical protein	3.11		Inner membrane
	GSU3559		Hypothetical protein	3.49		Non-cytoplasmic

**TABLE 2:** Downregulated genes in Co^II^ transcriptome.

Function (no. of genes)	Locus	Gene	Gene product	log(2) FC	Metal-binding motif (Pfam)	Subcellular localization
Folding, secretion, and degradation (1)	GSU0781	*fdnT*	Twin-arginine translocation pathway protein, TatA/TatE family	−1.95		Inner membrane
Carbohydrate metabolism (3)	GSU0778	*fdnH*	Periplasmically oriented, membrane-bound formate dehydrogenase, iron-sulfur cluster-binding subunit	−2.23	Two [4Fe-4S]-binding (PF13247, PF12800)	Inner membrane
	GSU0779	*fdnI*	Periplasmically oriented, membrane-bound formate dehydrogenase, b-type cytochrome subunit, putative	−2.02	NrfD, polysulfide reductase (PF03916)	Inner membrane
	GSU3125	*mtd*	mannitol dehydrogenase	−2.90	Zn^II^-binding dehydrogenase (PF00107)	Cytoplasm
Energy metabolism (2)	GSU1640	*cydA*	cytochrome bd menaquinol oxidase, subunit I	−3.13	Cytochrome bd terminal oxidase subunit I (PF01654)	Inner membrane
	GSU1641	*cydB*	cytochrome bd menaquinol oxidase, subunit II	−3.19	cytochrome bd terminal oxidase subunit II (PF02322)	Inner membrane
Cell redox homeostasis (2)	GSU3126		oxidoreductase, aldo/keto reductase family	−3.08		Cytoplasm
	GSU3246	*prx-2*	peroxiredoxin, typical 2-Cys subfamily	−2.63		Cytoplasm
Hydrolases (2)	GSU1159		Intracellular protease, PfpI family, putative/type 1 glutamine amidotransferase	−2.03		Cytoplasm
	GSU3122		Metal-dependent hydrolase, beta-lactamase superfamily	−2.14		Cytoplasm
Signal transduction (2)	GSU1639	*rrf2*	Winged helix-turn-helix transcriptional regulator, Rrf2 family	−3.45	Fe^II^ dependent transcriptional regulator (PF02082)	Cytoplasm
	GSU1643		Response receiver-modulated diguanylate cyclase	−1.99		Cytoplasm
DNA binding, replication, repair (1)	GSU3245		DNA polymerase II, putative	−2.69		Cytoplasm
Unknown function (2)	GSU0208		Hypothetical Protein/DUF4350 domain-containing protein	−2.97		Inner membrane
	GSU1160		Hypothetical protein	−2.14		Non-cytoplasmic

#### Periplasmic Detoxification of Co^II^

**FIGURE 5:**
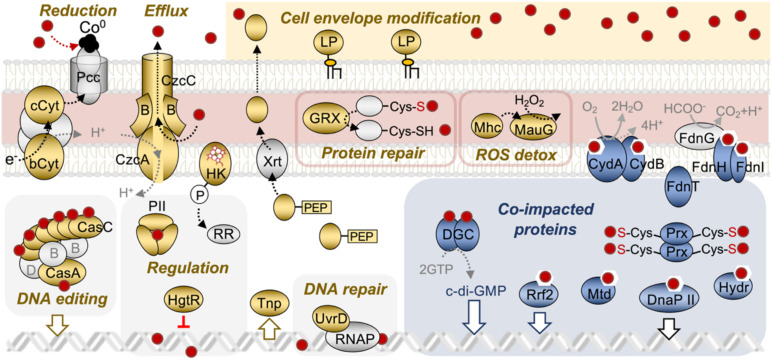
Model for the transcriptional response of *G. sulfurreducens* during growth under Co^II^ stress. The figure illustrates pathways (upregulated, gold; downregulated, blue) differentially expressed under Co^II^ stress. Proteins in gray represent proteins predicted to participate in the detoxification pathways that did not undergo differential expression. Additional information for the proteins and encoding genes is available in Tables 1, 2. bCyt, Cytochrome *b*; CasABCD, CRISPR-Cas complex; b/cCyt, Cytochrome *b* or *c*; CydAB, Cytochrome *bc* oxidase complex; DGC, diguanylate cyclase; FdhT, formate dehydrogenase chaperone; FdnGHI, formate dehydrogenase; GRX, glutaredoxin; HgtR, hydrogen-dependent growth transcriptional regulator; HK, heme-binding histidine kinase; Hydr, Hydrolase; LP, lipoprotein; MauG, MauG-like diheme peroxidase; Mhc, monoheme cytochrome *c*; Mtd, mannitol dehydrogenase; PII, P-II family nitrogen regulator (PII-NG); Pcc, Porin-cytochrome complex; RNAP, RNA polymerase; UvrD, UV repair protein D (DNA helicase of the nucleotide excision repair pathway); Xrt, exosortase.

The diffusion of Co^II^ through non-selective outer membrane porins (Nikaido, 2003) leads to its rapid accumulation in the periplasmic space and risks disruption of essential cellular functions such as protein secretion and respiration. Co^II^ toxicity in the periplasm is consistent with the upregulation of two periplasmic cytochromes (GSU1538 and GSU2513) with predicted roles in hydrogen peroxide (H_2_O_2_) detoxification (Figure 5). This suggests that Co^II^ accumulated in the periplasm at levels high enough to catalyze Fenton-chemistry reactions yielding reactive oxygen species (ROS) (Barras and Fontecave, 2011). GSU1538 has the conserved domain of di-heme cytochrome *c* peroxidases (PF03150), a group of periplasmic enzymes that reduce H_2_O_2_ to prevent oxidative stress (Pettigrew et al., 2006). Bacterial cytochrome *c* peroxidases can receive electrons from small monoheme cytochromes (Pettigrew et al., 2006). The upregulation of GSU2513, a periplasmic monoheme cytochrome *c* protein, suggests a similar redox partnership with the GSU1538 peroxidase.

Co^II^-stressed cells also upregulated GSU2812, a glutaredoxin-family protein (glutaredoxin motif, PF00462) containing a signal peptide (amino acids 1–27) for export to the periplasm. Glutaredoxins, like thioredoxins, are thioldisulfide oxidoreductases that reduce or oxidize disulfide bonds depending on the redox potential of the cellular compartment (cytoplasm or periplasm) where they operate (Eser et al., 2009). For example, *E. coli* secretes several thioredoxin proteins (e.g., DsbA and DsbC) to the periplasm to form disulfide bonds and fold proteins (Hatahet et al., 2014). A periplasmic monothiol glutaredoxin (glutaredoxin 3, Grx3) complements the activities of DsbA and DsbC in reactions dependent on the glutathione biosynthetic pathway (Eser et al., 2009). The high affinity of Co^II^ for thiol groups in cysteines leads to the rapid oxidation of the amino acid and the formation of non-native disulfide bonds, which glutaredoxins can resolve to prevent protein inactivation (Hiniker et al., 2005).

We also identified among the upregulated genes an operon containing the three subunits of one of the four RND systems (GSU2135–2137) identified in the genome of *G. sulfurreducens* (Figure 2). This RND transporter has a membrane-bound metal pump (GSU2135) homologous to CzcA from *Cupriavidus metallidurans* strain CH34 and CusA from *E. coli* (Kim et al., 2011). The pump binds the metal in the periplasm and undergoes conformational changes that move one proton into the cytoplasm and translocate the metal through a channel formed by the B and C subunits (Figure 5). CusABC complexes often work coordinately with periplasmic metal chaperones (CusF) to transport monovalent cations (Cu^I^ and Ag^I^). The lack of CusF chaperones in the *G. sulfurreducens* genome suggests that the Co^II^ RND transporter is a CzcABC system. Indeed, CzcABC transporters receive their name for their ability to mobilize the divalent cations cobalt, zinc, and cadmium (Kim et al., 2011). Furthermore, this metal efflux system plays roles in Co^II^ detoxification and resistance in other bacteria (Ma et al., 2020). Thus, we designated the GSU2135–2137 genes as *czcABC* (Table 1).

#### Cytoplasmic Detoxification of Co^II^

The first gene in the *czcABC* operon (GSU2134) codes for a protein with the conserved P_*II*_ domain (PF00543) of nitrogen regulatory proteins. These proteins form homotrimers to bind metabolites signaling the energy (ATP, ADP), carbon (2-oxogluratate) and nitrogen (glutamine and 2-oxoglutarate) levels inside the cell (Huergo et al., 2013). GSU2134 belongs to a phylogenetically distinct clade of proteobacterial P_*II*_ proteins (PII-NG) that evolved from the canonical nitrogen regulators GlnB and GlnK (Sant’Anna et al., 2009). Like most of the PII-NG proteins (Sant’Anna et al., 2009), GSU2134 clusters in the genome with the genes encoding a proton-cation CzcABC antiporter. Furthermore, PII-NG is a structural homolog of the metal-binding protein CutA1 of *E. coli* (Arnesano et al., 2003). CutA1 binds the divalent copper cation (Cu^II^) at a site structurally equivalent to the ATP binding site of PII-NG proteins (Arnesano et al., 2003) and uses metal binding to regulate genes involved in Cu^II^ tolerance (Fong et al., 1995). The structural homology of PII-NG and metal sensors together with its cytoplasmic location are consistent with a role in intracellular Co^II^ sensing and modulation of the regulatory cascade needed for cell acclimation to metal stress.

#### Indirect Effects of Co^II^ Stress on Fe^II^ Homeostasis

In *G. sulfurreducens*, the operon encoding the PII-NG regulator (GSU2134) and CzcABC proton/metal antiporter (GSU2135–2137) is also upregulated under Fe^II^ limitation via the master regulon of Fe^II^ homeostasis, Fur (Embree et al., 2014). To test if Co^II^ intoxication could indirectly limit the availability of Fe^II^, we used published transcriptomic data for *G. sulfurreducens* grown with sufficient versus excess Fe^II^ (Embree et al., 2014) to identify genes differentially expressed under Fe^II^ intoxication. More than half of the genes responding to Co^II^ stress (24 in total) were also differentially expressed during Fe^II^ intoxication (Figure 4C). However, most of the genes had opposite patterns of expression, supporting the idea that Co^II^ intoxication limits Fe^II^ availability. For example, a cluster of Fur-regulated genes comprised of the PII-NG-*czcABC* operon (GSU2134–2137) and upstream genes (GSU2129 and GSU2131–33) were upregulated by Co^II^ stress but downregulated in cells growing with excess Fe^II^.

We also identified a protein (GSU1639) with the conserved Rrf2 domain (PF02082) of Fe^II^-dependent transcriptional regulators (Keon et al., 1997; Schwartz et al., 2001) that was downregulated under Co^II^ stress but upregulated during Fe^II^ intoxication (Figure 4C). The Rrf2 domain ligates Fe or Fe-S clusters via redox-sensitive cysteine residues to tune the protein’s DNA specificity to Fe^II^ homeostasis (Rajagopalan et al., 2013). For example, the Rrf2 domain of *E. coli* IscR has three cysteines and one glutamic acid that bind Fe-S clusters to regulate genes involved in Fe-S cluster biosynthesis as a function of Fe^II^ availability (Schwartz et al., 2001). GSU1639 shares 55% similarity (33% identity) with IscR and has the conserved cysteines and glutamic acid needed for Fe-S cluster coordination at the Rrf2 domain. Furthermore, it is under direct control of Fur, the master regulator of Fe^II^ homeostasis (Embree et al., 2014). This suggests that GSU1639 binds Fe-S clusters to co-regulate the cluster’s biosynthetic pathways to Fe^II^ homeostasis. Co^II^ infiltration in Fe-S clusters and/or its high affinity for the cysteines in the Rrf2 binding site could prevent the regulator from sensing the Fe-S cluster signal and impair the ability of the cells to sense Fe^II^ availability.

Co^II^ but not Fe^II^ toxicity upregulated the hydrogen-dependent growth transcriptional repressor HgtR (GSU3364), a master regulator of central metabolism (Figure 4C). HgtR downregulates genes involved in energy generation and biosynthesis such as *gtlA* (citrate synthase in TCA cycle), *atpG* (ATP synthase F0 β’ subunit), and *nuoA* (NADH dehydrogenase I, A subunit) to tune growth rates to the cell’s nutritional status (Ueki and Lovley, 2010). The overexpression of the repressor provides a mechanism to adjust growth to the energy demands of cells coping with Co^II^ intoxication and low Fe^II^ availability. Fe^II^ limitation may have also triggered the induction of GSU0356, a heme-binding sensor histidine kinase that could regulate the cellular response to the accumulation of metal-free or Co^II^-impacted heme groups (Table 1). This histidine kinase lacks a signal peptide but contains three internal helices for insertion in the inner leaflet of the inner membrane, a subcellular localization optimal for cytoplasmic sensing. In addition to phosphoacceptor (HisKinA, PF00512) and ATPase (HATPase, PF02518) domains, the GSU0356 histidine kinase has a domain of unknown function (DUF3365, PF11845) with a heme-binding site (CXXCH sequence). Heme-responsive histidine kinases typically bind the heme group reversibly (Girvan and Munro, 2013). This sensory capacity allows the cells to prevent the toxic build-up of metal-free hemes (Dailey et al., 2017). The upregulation of the heme sensor during Co^II^ and Fe^II^ intoxication (Figure 4C) suggests that both conditions may have resulted in heme toxicity.

#### Evidence for DNA Damage

Co^II^-stressed cells upregulated components of one of the two Type I CRISPR loci (CRISPR2) in *G. sulfurreducens* (GSU1385 and GSU1387) (Table 1). The CRISPR2 locus (GSU1384–1393) contains 8 CRISPR-associated (Cas) proteins and an array with 143 spacers. The locus lacks a Cas4 protein but has Cse1 and Cse2 (named after the CRISPR system of *E. coli*) components, meeting the criterion for classification as a subtype I-E CRISPR (Makarova and Koonin, 2015). Co^II^-stressed cells upregulated Cse1 (GSU1385, also known as CasA), the large subunit of the antiviral defense Cas complex (Cascade) that facilitates RNA-guided recognition of complementary DNA (Makarova and Koonin, 2015). Cse1 recognizes a short protospacer adjacent motif (PAM) in the crRNA and discriminates self from foreign DNA targets (Westra et al., 2013). A Zn-finger motif in Cse1 binds Zn^II^ to control interactions with the target DNA (Gorelsky et al., 2005). This structural motif is sensitive to infiltration by Co^II^, a metal that changes the selectivity of the Cas complex for the target DNA and stimulates its nicking activity (Sundaresan et al., 2017). Co^II^ also upregulated Cse4 (GSU1387, also known as CasC or Cas7), a protein that polymerizes as a hexameric arch along the spacer region of the crRNA within the Cascade complex (Zheng et al., 2020). Cse4 has a ferredoxin-like fold in its RNA recognition motif (Makarova and Koonin, 2015) with a conserved metal-binding βαββαβ fold that could bind Co^II^ (Waldron and Robinson, 2009; Smith et al., 2015). The final result is a Co^II^-compromised Cascade complex with increased nicking activity that could lead to DNA damage. Co^II^ can also infiltrate the DNA helix and cause structural changes and breaks in the strands (Kanellis and Dos Remedios, 2018). Consistent with the need to repair damaged DNA, cells upregulated a UvrD helicase (GSU0763) of the nucleotide excision repair pathway. UvrD can also bind RNA polymerase (RNAP) stalled on the DNA lesions and backtrack the enzyme to expose the damaged site to DNA repair proteins (Epshtein et al., 2014). This mechanism allows the RNAP to resume transcription as soon as the repair has concluded.

#### Downregulation of Non-essential Metalloproteins

Most of the downregulated genes encoded non-essential proteins with prosthetic groups, metal-binding motifs and amino acids sensitive to Co^II^ inactivation (Table 2). Almost all of the targets where cytoplasmic or periplasmic redox-active proteins with Fe^II^-prosthetic groups (e.g., hemes and Fe-S clusters) or proteins with thiol-containing cysteines that Co^II^ readily binds and inactivates (Figure 5). For example, metal-stressed cells downregulated the two subunits (CydAB) of the cytochrome *bd* complex (GSU1640–1641), a respiratory quinol:O_2_ oxidoreductase widespread in prokaryotes that conserves energy from the transfer of electrons from the menaquinone pool to O_2_ (Borisov et al., 2011). Thus, the CydAB complex is not needed under the strictly anaerobic conditions used in our study. Similarly, cells downregulated two subunits (GSU0778–0779) of the trimeric formate dehydrogenase enzyme, FdnGHI, and the associated secretory protein FdnT (GSU0781), which are only needed when growing with formate as electron donor. Another example of a downregulated protein is Prx-2 (GSU3246), a cytoplasmic thioredoxin peroxidase of the 2-cysteine peroxiredoxin subfamily (Poole and Nelson, 2016). These thiol-based peroxidases scavenge the low levels of H_2_O_2_ produced intracellularly during normal growth and transduce the H_2_O_2_ signal to control cellular homeostasis (Toledano and Huang, 2016). When hyperoxidized, however, the enzymes aggregate and become chaperone holdases to protect proteins from denaturation (Toledano and Huang, 2016). The affinity of Co^II^ for the thiol groups of peroxiredoxins could impair these functions. Thus, cells downregulate its expression to minimize negative impacts of metal inactivation on the cellular stress response.

### Reductive Precipitation of Co^II^ as a Detoxification Mechanism

**FIGURE 6:**
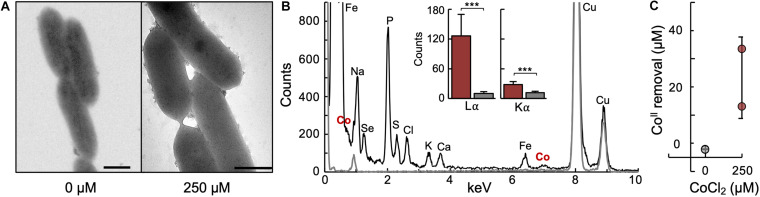
Co^II^ mineralization at the cell outer surface. **(A)** Transmission electron microscopy (TEM) images of unstained cells from untreated (0 μM CoCl_2_) and treated (250 μM CoCl_2_) cultures. Scale bar, 500 nm. **(B)** Representative X-ray energy dispersive (EDS) spectra of cells from 250 μM CoCl_2_ cultures (black) and cell-free control areas examined by TEM identifying Co energy signatures from the cells. The inset shows the average energy intensity (counts) detected for the primary X-ray Co emission peaks (Lα, 0.776 keV; Kα, 6.924 keV) for four different cells (maroon) and control (gray) samples. Pairwise comparisons (*t*-test) between the cell and cell-free Lα and Kα average intensities produced *p* values below 0.0001 (***). **(C)** Co^II^ removal by cells in cultures with or without 250 μM CoCl_2_ supplementation. The initial and final (early stationary phase) Co^II^ concentrations in culture supernatant fluids were calculated colorimetrically after complexation with 2-beta-mercaptoethanol and using a standard curve (linear fit from 0 to 500 μM CoCl_2_; *R*^2^ = 0.9947). The difference between the final and initial concentration of Co^II^ estimated the amount of metal removed by the cells.

We identified among the most upregulated genes two cytochrome-encoding genes (GSU0593 and GSU0594) that could participate in the extracellular reduction of Co^II^. GSU0593 and GSU0594 are the cytochrome *b* (CbcB) and cytochrome *c* (CbcA) subunits, respectively, of the Cbc5 menaquinol:ferricytochrome *c* oxidoreductase, a pentasubunit complex expressed during the reduction of Fe^III^ oxide minerals (Aklujkar et al., 2013). The cytochromes (*b* and *c1*) and Fe-S proteins in cytochrome *bc* complexes transfer electrons from the menaquinone pool via a proton motive Q-cycle pathway (Trumpower, 1990). To complete the Q cycle, Cbc5 catalyzes two “redox turnovers” that consume two protons in the cytoplasm and release four protons in the periplasm. Thus, each Q cycle transfers two electrons and contributes two protons to the transmembrane proton gradient. Spanning the inner and outer membranes, the Cbc5 complex could electronically connect the menaquinone carriers with *Geobacter* outer membrane porin-cytochrome *c* complexes (Pcc) (Shi et al., 2016). Given the known role of Pcc complexes in the reductive precipitation of some divalent cations to their elemental, metallic form (Hu et al., 2013), we examined untreated and Co^II^-treated cells by transmission electron microscopy (TEM) for the extracellular formation of metal nanoclusters (Figure 6A). To prevent artifactual mineralization of heavy metal salts used to negatively stain cells for TEM (Brenner and Horne, 1959), we examined unstained cells. This approach allowed us to visualize numerous electron dense nanoparticles on the surface of Co^II^-stressed cells that were absent in untreated samples (Figure 6A). The homogenous dispersal of the nanoclusters is consistent with the distribution of outer membrane cytochrome foci in *G. sulfurreducens* (Shi et al., 2016). Further, X-ray energy dispersive spectroscopy (EDS) analyses confirmed the presence of Co on the outer surface of the treated cells but not in cell-free controls, consistent with the immobilization of the metal on the cell surface (Figure 6B). Using a colorimetric assay based on the color response of Co^II^ when bound by 2-beta-mercaptoethanol (Figure 6B), we estimated that Co^II^-stressed cells had removed from the solution an average of 25 μM of the metal (Figure 6C). These results suggest that the detoxification response of *G. sulfurreducens* also included pathways for Co^II^ mineralization, as reported for the uranyl cation (Cologgi et al., 2011).

### Cell Envelope Modifications to Prevent Co^II^ Infiltration and Form Biofilms

The transcriptomics analyses identified lipoproteins (GSU2133 and 3576) and EPS-associated proteins (GSU1079 and GSU1994) that could have modulated the properties of the cell surface to prevent metal infiltration and promote its extracellular immobilization (Cologgi et al., 2014). At least one of the lipoproteins was predicted to be targeted to the outer membrane, the other one could only be confirmed as non-cytoplasmic (Table 1). Lipid modification of exported hydrophilic proteins facilitates their anchoring to the inner leaflet of the outer membrane yet most, if not all, of the lipoproteins get translocated to the outer leaflet (Schulze and Zuckert, 2006; Konovalova and Silhavy, 2015). Surface-exposed lipoproteins control the permeability of the cell to soluble substrates and can also mediate cell adhesion (Konovalova and Silhavy, 2015). Additional modifications to the cell envelope are expected from the upregulation of two proteins (GSU1079 and GSU1994) carrying the PEP-CTERM motif (PF07589) of EPS-associated proteins (Haft et al., 2006). The motif comprises a carboxy-terminal (CTERM) Pro-Glu-Pro (PEP) recognition peptide, a transmembrane helix and an arginine-rich cluster (Haft et al., 2006). The protein sorting signal is recognized and cleaved by a dedicated exosortase (Xrt) in the inner membrane and the mature protein is then exported to the EPS matrix by yet unknown secretory pathways (Haft et al., 2006). Except for the presence of the conserved sorting signal, PEP-CTERM proteins have little homology to other proteins. Most are, however, rich in serine and threonine, suggesting they are glycosylated during export (Haft et al., 2012). The genome of *G. sulfurreducens* encodes 5 PEP-CTERM proteins, an EpsH family Xrt exosortase (GSU1979) and a two-component system (PrsK histidine kinase, GSU1941; PrsR response regulator, GSU1940) predicted to regulate export (Haft et al., 2006).

**FIGURE 7:**
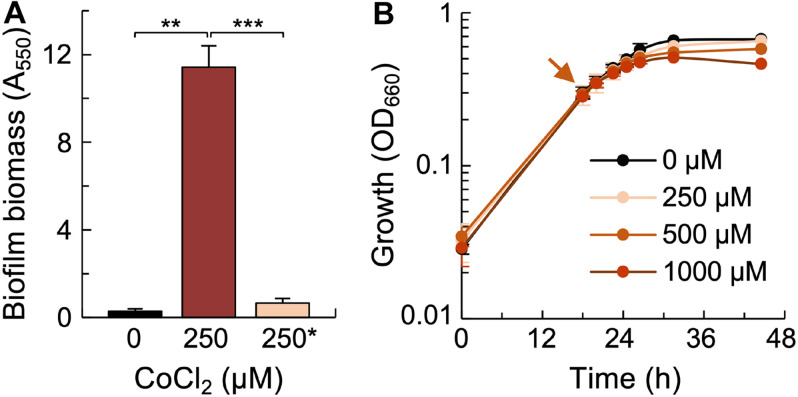
Adaptive responses of cells to Co^II^ stress. **(A)** Biofilm formation (absorbance at 550 nm of biofilm-associated crystal violet) in stationary phase cultures growing with 250 μM CoCl_2_ compared to the lack of biofilms in cultures growing without CoCl_2_ supplementation (0 μM) or challenged with the CoCl_2_ in mid-log phase (∼0.3 OD_660_) (250^∗^). Statistically significant differences in pairwise comparisons (*t*-test) are highlighted with asterisks (^∗∗^*p* < 0.0002 or ^∗∗∗^*p* < 0.0001). **(B)** Planktonic growth of cultures challenged with increasing concentrations of CoCl_2_ (arrow) in mid-log phase (∼0.3 OD_660_), including the ones used for the biofilm assays shown in panel **(A)** (250^∗^).

The widespread presence of PEP-CTERM proteins in Gram-negative bacteria that form biofilms suggests a role for these proteins in the development of surface-attached communities (Haft et al., 2006). In support of this, the expression of EPS-associated proteins in Co^II^-stressed cells of *G. sulfurreducens* preceded the formation of thick biofilms at the bottom of the tube once the cultures reached stationary-phase (Figure 7A). We did not identify in the transcriptome any of the genes that encode proteins required for the synthesis of the biofilm EPS, Xap (Rollefson et al., 2011). This is not unexpected, because *G. sulfurreducens* expresses the *xap* genes during exponential growth in acetate-fumarate cultures (Rollefson et al., 2011). The Xap EPS anchors outer membrane cytochromes (Rollefson et al., 2011) and provides a mechanical and redox barrier to the permeation of soluble divalent metal cations in biofilms (Cologgi et al., 2014). To test for a similar protective effect by the EPS produced by planktonic cells, we challenged mid-log phase cultures with up to 1 mM concentrations of CoCl_2_ and monitored the effect of the metal treatments in growth (OD_6__6__0_). As shown in Figure 7B, metal shock had little effect on growth efficiency in any of the cultures. Such high levels of metal resistance are consistent with the role of the EPS matrix in preventing metal permeation. Furthermore, planktonic cultures challenged with the metal did not form thick biofilms in stationary phase (Figure 7A). Thus, biofilm formation appears to be an adaptive response to persistent metal toxicity.

## Discussion

The high Co^II^ tolerance and complex acclimation response of *G. sulfurreducens* is consistent with selection mechanisms during long-term environmental exposure to the metal. Fe^III^ and Mn^IV^ oxides form heterogenous mixes with natural organic matter and metal micronutrients (Huang and Zhang, 2020) that provide optimal conditions for the growth of *Geobacter* species (Reguera and Kashefi, 2019). The high reactivity of the Fe^III^ and Mn^IV^ (hydr)oxides sequesters Co^II^ and other metal cations in the mineral phases (Backes et al., 1995; Krupka and Serne, 2002), concentrating them in the metal oxide-rich epigenetic zones (Burkhardt et al., 2009). The reductive dissolution of the metal-bearing minerals mobilizes the metal cations (Huang and Zhang, 2020) and increases their concentration in the pore-water to toxic levels (Weber et al., 2009). Cu^II^, for example, can be mobilized to levels (∼20 μM) above the minimum concentration (10 μM) that inhibits the growth of *G. sulfurreducens* in the laboratory (Kimber et al., 2020). Yet, this bacterium grew from low cell densities, albeit with trade-offs in growth efficiency, in the presence of up to 1 mM CoCl_2_ (Figure 3). Furthermore, it was relatively unaffected when exposed to the same metal concentrations during the exponential phase of growth (Figure 7). We attributed this to the expression in exponentially growing cells of the biofilm EPS (Rollefson et al., 2011), which can shield the cells from metal infiltration. Cell density can also affect cellular metabolism and the secretion of metabolites that change the chemical speciation, bioavailability, and toxicity of metals (Franklin et al., 2002). Furthermore, increases in cell numbers activate stress responses that acclimate the population and increase tolerance to a number of stressors (Li et al., 2001). By contrast, cells inoculated at low densities must first reprogram their physiology to acclimate to and initiate growth in the presence of the metal stressor. Acclimation is evident in the extended periods of growth arrest (*lag* phase) that *G. sulfurreducens* cultures initially experienced when growing with sublethal concentrations of CoCl_2_ (Figure 3) and in the multiple cellular pathways that were activated to couple growth to Co^II^ detoxification (Figure 4).

The transcriptomic studies provided insights into the extensive transcriptional reprogramming that allowed *G. sulfurreducens* to cope with Co^II^ stress (Figure 5). Transcript levels for Co^II^ importers remained constant, consistent with the absence in *G. sulfurreducens* of transcriptional regulators (e.g., CzrA and CoaR) that other bacteria use to directly control Co^II^ uptake for metal homeostasis (Waldron and Robinson, 2009). Instead, *G. sulfurreducens* acclimation involved metal (PII-NG) and heme (GSU0356 histidine kinase) sensors and a transcriptional regulator of central metabolism (HgtR) (Figure 5). Cells also upregulated a CzcABC pump for proton-driven export of metal traversing the outer membrane, a canonical mechanism used by other Gram-negative bacteria to increase metal resistance (Ma et al., 2020). In addition, Co^II^ upregulated a periplasmic glutaredoxin, which repairs and rejoins cysteines oxidized by Co^II^ to refold proteins to their native and functional conformation (Ezraty et al., 2017). The activation of a periplasmic MauG-like di-heme cytochrome *c* peroxidase (GSU1538) suggested that Co^II^ accumulated in the periplasm at levels sufficiently high to generate H_2_O_2_ (Barras and Fontecave, 2011). Di-heme cytochrome *c* peroxidases detoxify H_2_O_2_ in the periplasm by reducing it to two H_2_O molecules (Pettigrew et al., 2006). This reaction receives electrons from a dedicated electron donor such as the monoheme cytochrome GSU2513, which was also upregulated by Co^II^ (Table 1). Without the peroxidase-cytochrome pair, H_2_O_2_ would oxidize solvent-exposed [4Fe-4S]^2+^ clusters in proteins, producing inactive [3Fe-4S]^3+^ species that abolish the redox activity of the metalloprotein (Imlay, 2008). The detoxification of H_2_O_2_ is also important to prevent Fenton-like reactions that generate highly toxic ∙OH radicals and exacerbate oxidative stress (Leonard et al., 1998).

Despite mechanisms for periplasmic detoxification, Co^II^ may have infiltrated the cytoplasm and damaged essential macromolecules. The presence of cytoplasmic chelators such as glutathione facilitates reactions between Co^II^ and H_2_O that generate ROS and oxidatively damage DNA (Leonard et al., 1998). Co^II^ can also bind components of the CRISPR Cascade complex that mediates antiviral defense, changing its specificity for target DNA and stimulating its RNA-independent DNA cleavage activity (Sundaresan et al., 2017). To cope with DNA damage, *G. sulfurreducens* activated the expression of UvrD, a helicase of the nucleotide excision repair pathway (Kamarthapu and Nudler, 2015) and transcription-coupled repair (Epshtein et al., 2014). The latter is particularly important to maintain the transcriptional activity of the cell during metal intoxication. This is because UvrD associates with NusA to backtrack RNAP when stalled at a DNA lesion. The helicase then recruits the UvrAB repair complex to the damaged site (Epshtein et al., 2014). This intervention allows the RNAP to resume transcription as soon the lesion is repaired (Kamarthapu and Nudler, 2015).

The Irving-Williams series (Mn^II^ < Fe^II^ < Co^II^ < Ni^II^ < Cu^II^ > Zn^II^) predicts greater stability for Co^II^ than Fe^II^ or Mn^II^ complexes independently of the ligand (Hill and Sadler, 2016). As a result, Co^II^ intoxication preferentially impacts Fe^II^ and Mn^II^ metalloproteins. To prevent the retention of the toxic metal in the metalloproteome, *G. sulfurreducens* downregulated non-essential proteins with Fe^II^ prosthetic groups (Figure 5). Nearly all of the downregulated proteins contained Fe-S clusters or metallocenters coordinating Fe^II^ atoms (Table 2). The chemical similarities with Fe^II^ facilitate the infiltration of Co^II^ into Fe-S clusters but the greater electron density of Co^II^ alters the coordination of the metal with the enzyme and its activity (Thorgersen and Downs, 2007; Waldron and Robinson, 2009). Co^II^ is also able to compete with Fe^II^ for binding to the porphyrin ring of heme groups such as those in cytochromes (Thorgersen and Downs, 2007). This could be catastrophic in the periplasm, where heme-containing respiratory chains are particularly abundant. Co^II^-hemes are weaker transporters of charges than the native Fe^II^-hemes (Majtan et al., 2011), impairing, or even abolishing, respiratory growth. To compensate for this, *G. sulfurreducens* downregulated non-essential heme-containing proteins such as the cytochrome *bd* oxidase subunits CydAB required for aerobic respiration (Figure 5). Similarly, cells downregulated genes encoding the formate dehydrogenase complex (the Fe-S cluster protein FdnH and the cytochrome *b* FdnI) and the secretory accessory protein FdnT, as these proteins are only needed for formate-dependent growth. Cells also downregulated an Rrf2 protein (GSU1639), which uses cysteine residues to bind Fe-S clusters and co-regulate Fe-S cluster biosynthesis and Fe^II^ homeostasis (Schwartz et al., 2001). The high affinity of Co^II^ for cysteines may prevent Rrf2 protein from sensing Fe-S cluster availability in the cytoplasm. To prevent further deregulation of Fe^II^ homeostasis, cells downregulated the *rrf2* gene (Table 2).

The principles of the Irving-Williams series (Hill and Sadler, 2016) also explain the high affinity of Co^II^ for Fe^II^-heme. Downregulating non-essential proteins with Fe^II^-hemes can provide some partial relief (Table 2). However, Co^II^ can also infiltrate the Fe^II^-hemes during their biosynthesis and prevent their incorporation into proteins. This leads to the accumulation of free Co^II^-hemes in the cytoplasm and cytotoxicity (Lin and Everse, 1987). The upregulation of a heme-containing histidine kinase (GSU0356) (Table 1) could provide a mechanism to sense the impact of Co^II^ on the heme pool and coordinate the heme detoxification response, as reported in other bacteria (Anzaldi and Skaar, 2010). The advantage of this heme-sensing mechanism is that cells can simultaneously co-regulate heme biosynthesis to Co^II^ and Fe^II^ homeostasis (Dailey et al., 2017). We initially reasoned that Co^II^ infiltration in the free hemes could have increased the intracellular levels of Fe^II^ and exacerbate metal toxicity (Lin and Everse, 1987; Anzaldi and Skaar, 2010). For example, free Fe^II^, like Co^II^, can generate ROS via Fenton chemistry and cause intracellular damage (Everse and Hsia, 1997). However, although Co^II^ and Fe^II^ intoxication had overlapping transcriptional responses, most of the shared gene targets were reversely regulated (Figure 4C). Thus, cells faced conditions of Fe^II^ limitation during Co^II^ intoxication. The accumulation of Co^II^ in the periplasm could competitively exclude Fe^II^ from import across the inner membrane, reducing its intracellular availability. Furthermore, once removed from metalloproteins and prosthetic groups, Fe^II^ can be sequestered non-specifically by cytoplasmic chelators, effectively reducing its intracellular availability.

In addition to mechanisms for metal detoxification in the periplasm and cytoplasm, *G. sulfurreducens* induced pathways that could have promoted the extracellular immobilization of the metal. For example, cells upregulated outer membrane lipoproteins that could have modulated the permeability of the outer membrane (Nikaido, 2003) and/or function as adhesins to promote cell-cell aggregation (Konovalova and Silhavy, 2015). Additionally, Co^II^ triggered the expression of EPS-associated proteins (PEP-CTERM proteins) typically expressed by biofilm-forming bacteria (Haft et al., 2006). The synthesis by planktonic cells of *G. sulfurreducens* of the biofilm EPS (Xap) precedes biofilm formation and allows the cell to anchor to the Xap matrix cytochromes needed for metal reduction (Rollefson et al., 2011). This redox activity could allow the planktonic cells to reductively precipitate Co^II^ on the cell surface, generating the metal nanoclusters visualized by TEM (Figure 6A). The mineral particles resolved by TEM formed on discreet foci on the cell surface, similarly to the distribution of outer membrane cytochromes of the Pcc complexes (Qian et al., 2007). Furthermore, the Pcc outer membrane cytochromes can bind and reductively precipitate divalent metal cations to their elemental form (e.g., Hg^II^ to Hg^0^) (Hu et al., 2013). A similar reaction could allow the cytochromes to reductively precipitate Co^II^ to Co^0^ on the cell surface. The Pcc outer membrane cytochrome complexes contain periplasmic and extracellular *c*-type cytochromes within an outer membrane porin to electronically connect periplasmic carriers to extracellular electron acceptors (Shi et al., 2016). The upregulation by Co^II^ of a respiratory cytochrome *bc* complex (Cbc5) could provide a mechanism for energy conservation from the reduction of Co^II^ at the Pcc foci (Figure 5). The Cbc5 complex is anchored to the inner and outer membranes and could interact with the periplasmic cytochrome of the Pcc complex to complete the electron transfer pathway to Co^II^ (Figure 5). Although none of the Pcc genes were differentially expressed by Co^II^, we confirmed the upregulation of the Pcc outer membrane *c-*cytochrome OmcC (GSU2731) when the false discovery rate (FDR) threshold was increased from 0.05 to 0.08. This could indicate that some cells may be upregulating the PccC cytochrome. Alternatively, cells may constitutively produce the Pcc complexes under the culture conditions used in our study. Experimental testing of this hypothesis is warranted.

The expression of lipoprotein adhesins and a redox-active EPS could also have allowed cells to aggregate and form biofilms (Figure 7A), an adaptive response that confers on *G. sulfurreducens* increased resistance to soluble, toxic metals (Cologgi et al., 2014). The downregulation of a cytoplasmic diguanylate cyclase (DGC) with a canonical GGDEF domain (GSU1643) (Table 2) in Co^II^-stressed cells may have reduced the intracellular levels of c-di-GMP in order to regulate the planktonic-to-biofilm transition. Most DGC enzymes contain sensory domains that modulate the synthesis of the bacterial second messenger bis-(3′,5′)-cyclic dimeric guanosine monophosphate (c-di-GMP) to specific input signals, including metals. For example, Zn^II^ reversibly binds the subunits of the *E. coli* DgcZ dimer (formerly YdeH) to allosterically regulate the synthesis of c-di-GMP (Zähringer et al., 2013). The *Geobacter* DGC enzyme does not have metal-binding domains but has instead the *N*-terminal phosphoreceiver (REC) domain of DGCs in the PelD superfamily (Table 2). The best studied PelD-like DGC is WspR, the response regulator of the Wsp chemosensory pathway that regulates cell-cell aggregation and biofilm formation in *Pseudomonas aeruginosa* (D’Argenio et al., 2002). Phosphorylation of the receiver domain in the WspR dimer activates the synthesis of c-di-GMP and autoaggregative/biofilm phenotypes (Hickman et al., 2005). Mg^II^ cations bind near the receiver’s active site of the WspR dimer and contribute to its activity (De et al., 2008). The downregulation in Co^II^-stressed cells of the DGC enzyme could reflect a feedback mechanism to the infiltration of Co^II^ in the protein (Waldron and Robinson, 2009). Alternatively, Co^II^-stressed cells may have downregulated the WspR-like DGC to reduce GTP demand for c-di-GMP and increase the availability of the nucleotide triphosphate for EPS synthesis (Rehm, 2010). The EPS matrix can then promote cell-cell aggregation and biofilm formation as a protective mechanism against metal toxicity (Cologgi et al., 2014).

Biofilm formation in *G. sulfurreducens* embeds the cells in an electroactive matrix of cytochromes and conductive pili that effectively immobilizes soluble metals (Cologgi et al., 2014). The conductive pili are particularly important to overcome metal toxicity in biofilms because they provide a large redox surface area for the extracellular immobilization and reductive precipitation of toxic metals (Cologgi et al., 2011, 2014). The pilus surface is decorated with specialized motifs optimal for the coordination of divalent metal cations (Feliciano et al., 2015). These metal traps have high affinity for Co^II^ and, at high enough potentials, can reductively precipitate it as Co^0^ nanoparticles (Cosert and Reguera, 2019). Furthermore, the conductive pili are retractable appendages (Speers et al., 2016), a dynamic feature that allows cells to detach the minerals and recycle the structural peptides in the membrane for a new cycle of pilus polymerization and metal reduction (Reguera, 2018). We did not identify in the Co^II^ transcriptome any of the genes encoding proteins of the pilus biosynthetic apparatus (Table 1) nor did we observe pilus filaments by TEM (Figure 6). This was not unexpected because we used growth temperatures (30°C) that prevent pilus assembly in planktonic cells (Reguera et al., 2005; Cologgi et al., 2011). Under these conditions, cytochrome respiratory chains involving outer membrane Pcc complexes provided the primary pathway for extracellular electron transfer in Co^II^-stressed cells. Thus, Pcc cytochromes could have promoted the mineralization of Co^II^ on discreet surface foci as a detoxification mechanism (Figure 6).

The presence of metal nanoclusters on the surface of Co^II^-treated cells suggests that hitherto unknown biological reactions could contribute to the geochemical cycling of this important metal. We estimated that, on average, cells removed from the solvent 25 μM concentrations of Co^II^ (Figure 6C). As a comparison, the intracellular Co^II^ quota is in the low to sub-μM range and typically below the limits of detection of mass spectrometry assays (Outten and O’Halloran, 2001). Co^II^ biomineralization may be more significant in biofilms, thanks to the concentration in the biofilm matrix of conductive pili (Cologgi et al., 2014; Steidl et al., 2016) with high affinity motifs for Co^II^ binding and reduction to Co^0^ (Cosert and Reguera, 2019). These adaptive responses confer on *Geobacter* a competitive advantage for growth in metal-rich environments despite the mobilization of Co^II^ during the reductive dissolution of metal oxide mineral phases. The ability of *Geobacter* bacteria to reductively precipitate Co^II^ could also alleviate metal stress on syntrophic partners that depend on interspecies cobamide transfer to sustain their metabolism. Furthermore, the formation of Co^0^ nanoparticles effectively metallizes the cell surface and could allow *Geobacter* cells to gain energy from the reduction of low potential electron acceptors and to transfer respiratory electrons to syntrophic partners. Hence, Co^II^ mineralization may help define the niche space of *Geobacter*-driven microbiomes and provide molecular markers to predict the impact of their activities in the fate of this and other essential elements.

## Materials and Methods

### Genomic Reconstruction of Pathways for Co^II^ Transport and Assimilation

We performed a literature survey and used the KEGG database and BLAST searches to identify genes in the *G. sulfurreducens* genome with a predicted role in Co^II^ homeostasis and assimilation into cobamide synthesis. The subcellular localization of the protein products was predicted with PSORTb 3.0 (Yu et al., 2010).

### Bacterial Strains and Culture Conditions

*Geobacter sulfurreducens* strain PCA was obtained from our laboratory culture collection and routinely maintained in anaerobic mineral medium DB with 20 mM acetate and 40 mM fumarate, as described elsewhere (Speers and Reguera, 2012). The cultures were incubated at 30°C while periodically monitoring growth as optical density at 660 nm (OD_660_). Unless otherwise indicated, culture transfers to fresh medium were in mid-log phase (OD_660_, 0.3–0.4) and diluting the cells to an initial OD_660_ of 0.03. When indicated, the media was supplemented with CoCl_2_ from stock anaerobic solutions of 5 and 50 mM CoCl_2_ prior to cell inoculation. For some experiments, CoCl_2_ was added to mid-log phase cultures (∼0.3 OD_660_) incubated at 30°C. All growth experiments were performed in triplicate cultures. Cultures that did not initiate growth for 7 days were discarded (routinely one out of three replicates grown from low cell densities with 500 and 1,000 μM CoCl_2_, as shown in Figure 3). Growth curves (OD_6__6__0_) for each of the replicate cultures were analyzed to calculate the length of the *lag* phase (time before start of exponential growth), generation (doubling) time in exponential phase, and biomass yields (OD_6__6__0_ reached once the cultures entered stationary phase). The latter was expressed as relative growth yield in the treated (with 100–1,000 μM CoCl_2_) versus the untreated (0 μM CoCl_2_) cultures.

### RNA Extraction and Sequencing (RNAseq)

Cells were grown to mid-log phase (∼0.3 OD_660_) in the presence or absence of sublethal concentrations of CoCl_2_ (250 μM) before adding 1 ml of water saturated phenol (5% [v/v] Ambion® water saturated phenol, pH 6.6 in ethanol) to stop transcription. We harvested the cells by centrifugation (3,800 rpm, 8 min, 4°C) and extracted their RNA with the QIAGEN RNeasy kit (QIAGEN) following manufacturer’s recommendations. DNA digestion in the RNA samples used the QIAGEN RNase-free DNase Set and was confirmed by Reverse Transcriptase (RT)-PCR (Verso 1-step kit, Ambion). After assessing RNA integrity in a BioAnalyzer 2100 (Agilent), we selected the samples with the best RNA quality (two biological replicates from each treatment, 0 or 250 μM CoCl_2_) for RNA sequencing at Michigan State’s Research Technology Support Facility (RTSF, Genomic core). The facility uses validated procedures for rRNA depletion, library preparation, and Illumina Hi-Seq 4000 sequencing. Briefly, rRNA depletion used an Illumina TruSeq Total RNA Library Preparation kit with QIAseq FastSelect – 5S/16S/23S rRNA depletion (QIAGEN). Libraries were quantified using Qubit and Advanced Analytical Fragment Analyzer High Sensitivity DNA NGS assays. The libraries were then pooled in equimolar amounts for multiplexed sequencing and the pool was quantified using the Kapa Biosystems Illumina Library Quantification qPCR kit. Sequencing was in one lane of the Illumina HiSeq 4000 flow cell in 1 × 50 bp single read format and using SBS reagents. Base calling was with the Illumina Real Time Analysis (RTA) v2.7.7 software while demultiplexing and conversion to FastQ format was with Illumina Bcl2fastq v2.19.1 package.

We analyzed the RNAseq data from the Co^II^-treated and untreated samples with the SPARTA pipeline (Johnson et al., 2016), using FastQC and Trimmomatic tools for quality control and trimming and Bowtie for sequence alignment to the reference genome (GCA_000007985.2 *G. sulfurreducens* PCA). Gene-level transcript level abundance was calculated with the HTSeq software while the *edgeR* tool provided the differential expression values. Data filtering used a FDR < 0.05, log CPM > 5, and a log_2_ FC < −1 (downregulated genes) or >1 (upregulated genes) (Johnson et al., 2016). We used the R software^1^ with *pheatmap* function to draw clustered heatmaps of differentially expressed genes. Individual searches in BioCyc 24.0 (Karp et al., 2019) predicted the operon organization of the genes and identified one gene in an RND efflux pump operon (GSU2137) that did not make the maximum FDR value yet met the log CPM and fold-change thresholds. We added this gene to Table 1. We also searched each of the differentially expressed genes in the UniProtKB (UniProt Consortium, 2019) and KEGG databases to assign functional roles. The subcellular localization was predicted using the sequence analysis tools at UniProtKB (SignalP), PSORTb 3.0 (Yu et al., 2010) and CELLO v.2.5 (Yu et al., 2004). Predictions about the domain organization of each protein and identification of metal-binding motifs used the Pfam 33.1 (El-Gebali et al., 2019) tool available at the UniProtKB database. The curated RNAseq data (Supplementary Data 1) has been deposited in the Gene Expression Omnibus (GEO) functional genomics data repository^2^ under accession number GSE157146. We also identified in the RNAseq data genes differentially regulated in *G. sulfurreducens* under Fe^II^ intoxication, which were calculated as the ratio between Fe excess and homeostatic transcript abundance from microarray data reported elsewhere (Embree et al., 2014). The transcriptomic data comparisons used to generate a heatmap of Co^II^ versus Fe^II^ intoxication (Figure 4C) are provided as a Supplementary Data 2.

### Transmission Electron Microscopy (TEM) and X-Ray Energy-Dispersive Spectroscopy (EDS)

Mid-log phase cells from untreated or treated (0 or 250 μM CoCl_2_ supplementation, respectively) cultures were fixed with 2.5% glutaraldehyde before deposition for 5 min on Formvar-coated grids (150 square-mesh Ni, Electron Microscopy Sciences). After three washes with ddH_2_O (30 s each), we side-blotted the excess liquid and stored the samples at room temperature until ready for TEM examination. The cells were unstained to prevent stain and mineral artifacts during examination with a JEOL 1400 Flash 120kV transmission electron microscope. For EDS elemental analyses, we deposited Co^II^-stressed cells on a PELCO NetMesh^TM^ copper grid coated with a lacey formvar/carbon support film and examined the samples with a JEM-2200FS ultra-high resolution TEM instrument equipped with an EDS detector. To minimize interference during EDS detection, we collected energy spectra from cells exposed in the holes of the support film and, as controls, from similar areas within the grid that had no cells. We used the two primary X-ray signatures of Co around 0.776 keV (Lα peak) and 6.924 keV (Kα peak) to compare the average intensity of the cell-associated Co^II^ to the cell-free controls. Because the detection limit of the EDS system is 0.001 keV, we averaged the counts detected at 0.76, 0.77, and 0.78 keV to calculate the intensity of the Lα peak and of 6.91, 6.92, and 6.93 keV for the Kα peak. The peak intensities from cells and cell-free areas on the grid were statistically analyzed with the unpaired, unequal variance *t*-test function of the Excel software.

### Colorimetric Detection of Co^II^

We developed a colorimetric assay for the detection of Co^II^ in culture supernatant fluids based on the color response of the metal after complexation with 2-β-mercaptoethanol (BME). The reducing agent, BME, replaces the water molecules in the cobalt hexaaqua complex (Co[H_2_O]_6_)^2+^, turning the solution brown and permitting the spectrophotometric detection of Co^II^ at 475 nm. Prior to the assay, we grew *G. sulfurreducens* in DBAF medium at 30°C with or without 250 μM CoCl_2_ supplementation, collected 200-μl samples periodically, and recovered the culture supernatant fluids after centrifugation at 20,000 rcf for 3 min. To initiate the complexation reaction, we added 10 μl of BME (from a freshly prepared 100 mM aqueous stock) to 190 μl of supernatant sample. After mixing the solution by aspiration with a pipette, we incubated the reactions at 30°C for 20 min to reach their maximum color response and measured the absorbance at 475 nm against CoCl_2_ standards (0 to 500 μM CoCl_2_) prepared in DBAF medium. We calculated the amount of Co^II^ removed by the cells in the cultures as the difference between the initial and final concentration of the metal in the culture supernatant fluids.

### Biofilm Assay

Biofilm formation in stationary phase cultures was measured with a crystal violet assay (Merritt et al., 2005). Briefly, we poured out the liquid culture (∼ 10 ml) once the cells reached stationary phase, gently rinsed the tubes with ddH_2_O and added 1 ml of 0.1% crystal violet to stain the biomass of biofilms formed at the bottom of the tube. After 15 min, we poured out the crystal violet solution, rinsed the tubes with ddH_2_O and left them to dry overnight. We used 1 ml of 30% acetic acid to solubilize the biomass-associated crystal violet for 15 min and measured its absorbance at 550 nm to estimate the biofilm biomass.

## Data Availability Statement

The datasets presented in this study can be found in online repositories. The names of the repository/repositories and accession number(s) can be found in the article/Supplementary Material.

## Author Contributions

GR conceived and designed the study, with contributions from HD, MT, and KK to parts of the experimental design. GR, HD, and MT performed the genomic analyses. HD and MT performed the growth studies and cobalt assays. MT carried out the RNAseq experiments and GR and HD interpreted the results. HD performed the microscopy analyses, cobalt challenge, and biofilm assays. GR wrote the first draft of the manuscript. All authors contributed to manuscript revision and read and approved the submitted version.

## Conflict of Interest

The authors declare that the research was conducted in the absence of any commercial or financial relationships that could be construed as a potential conflict of interest.

## Footnotes

1 http://www.r-project.org

2 https://www.ncbi.nlm.nih.gov/geo/
